# A Novel Method of Object Detection from a Moving Camera Based on Image Matching and Frame Coupling

**DOI:** 10.1371/journal.pone.0109809

**Published:** 2014-10-29

**Authors:** Yong Chen, Rong hua Zhang, Lei Shang

**Affiliations:** School of Energy Science and Engineering, University of Electronic Science and Technology of China, Chengdu, China; Institute of Automation, Chinese Academy of Sciences, China

## Abstract

A new method based on image matching and frame coupling to handle the problems of object detection caused by a moving camera and object motion is presented in this paper. First, feature points are extracted from each frame. Then, motion parameters can be obtained. Sub-images are extracted from the corresponding frame via these motion parameters. Furthermore, a novel searching method for potential orientations improves efficiency and accuracy. Finally, a method based on frame coupling is adopted, which improves the accuracy of object detection. The results demonstrate the effectiveness and feasibility of our proposed method for a moving object with changing posture and with a moving camera.

## Introduction

Object detection has always been the key realm of CV (computer vision). In this field, object detection with a moving camera is a topic that has attracted many scholars. Methods used in this field can be divided into two categories, optical flow methods and motion compensation methods. Optical flow methods have been widely used in video surveillance with a stationary camera. However, this approach is complicated, to some extent, and is sensitive to the changing conditions of the background [1].

Motion compensation estimates the location of an object by calculating the motion vector; however, the motion vectors of a moving object and camera make it difficult to implement the process of motion compensation [2].

An approach based on macro block operation was presented [3]. This method calculates displacements and then integrates images with the same patterns. However, the accuracy of this method is low. Moreover, this approach is sensitive to changes of the moving object’s posture.

An algorithm based on HMM (Hidden Markov Model) is developed to describe the relationship between the moving object and the camera in the tracking system [4]. First, this method detects the object via color and optical flow information. Moreover, KLT (Kanade Lucas Tomasi) and a particle filter are used to implement the process of object tracking [5]. A new approach that simulates the process of foreground detection via the Bayes algorithm was proposed by Berrabah [6]. First, the approach models the background based on GMM(Gaussian Mixture Model). However, this method depends on parameters. In the tracking section, a particle filter is adopted to track the moving target, neglecting the motion parameters [7], [8]. A moving target detection approach combining SURF feature points and a data-clustering algorithm has been presented. First, this method extracts SURF feature points in each frame. Then, the k-means algorithm is used to implement the process of feature vector clustering between k frames [9].

**Figure 1:**
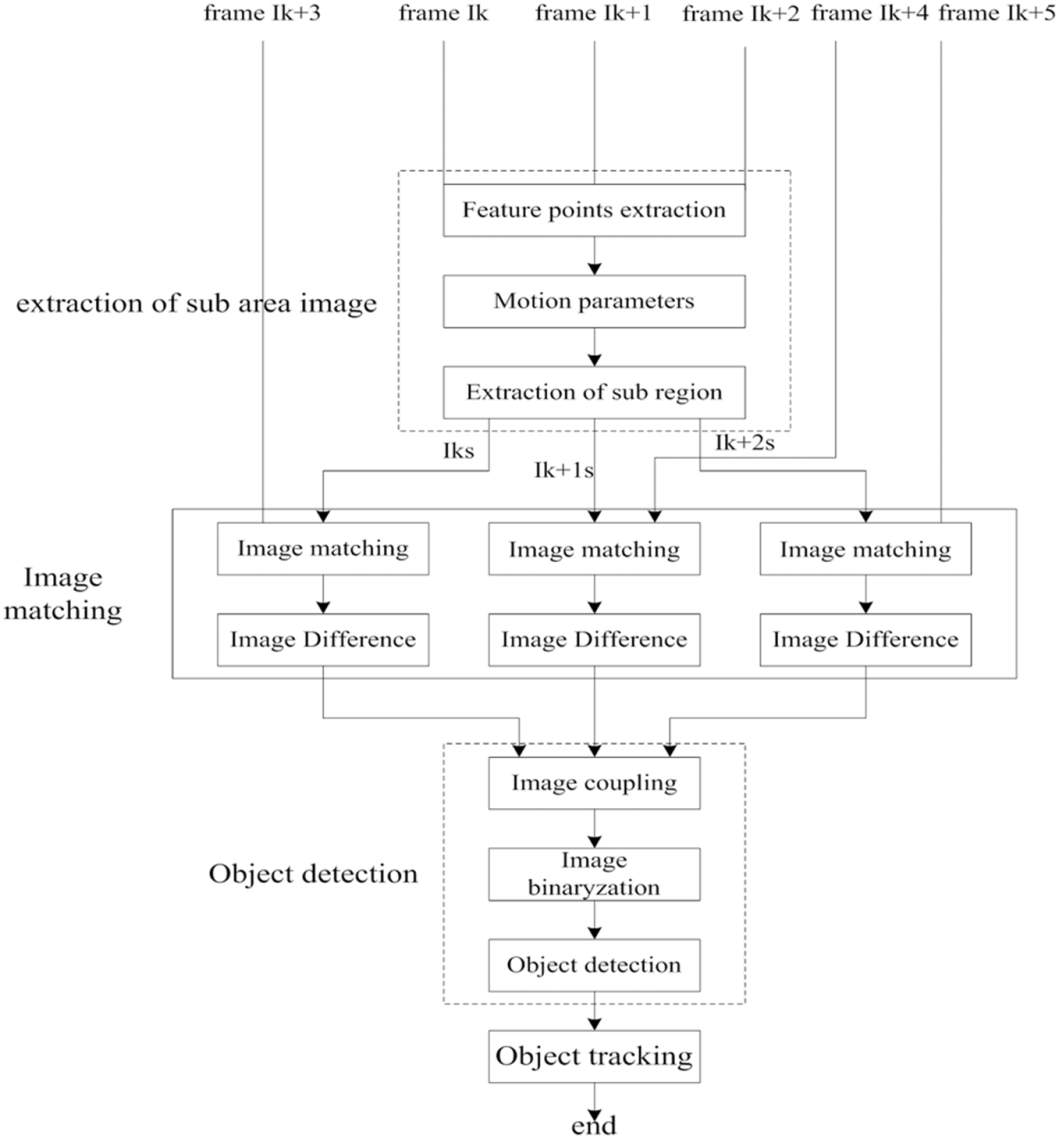
The framework of our method based on image matching and frame coupling.

To remedy the drawback of object detection caused by the motion of the camera and moving object, a new method based on image matching and frame coupling will be presented in this paper. First, this approach implements the process of image matching on scale-invariant feature points. Then, this method performs macro-block matching using the motion parameters of the camera. The basic process of this method is shown in Figure 1.

## Motion Parameters

With a moving camera, the frame sequences will be affected by the parameters of the camera. In this situation, this motion is called background motion. In video sequences, the changing of frame sequences caused by the motion of an object is called local motion. In our research, the camera is always changing. We can simulate the motion of camera via global motion and local motion and perform background compensating.

In the process of calculating parameters of the camera, we should focus on rotation and translation. In practical applications, a motion model with eight parameters can obtain more precise results. However, the process of obtaining parameters via this model is somewhat complicated. The affine transformation model has attracted more attention from researchers. This model can simulate rotation, scaling, translation and the corresponding Cartesian transformation. Because of its merits, we adopt the affine transformation model in our research. Using this model, we have to obtain three pairs of feature points to calculate six parameters. In this process, the least squares method is adopted to generate the motion parameters.

In the process of extracting feature points, corner points will not achieve satisfactory results due to the low precision of feature point locations. Moreover, considering the scaling and rotation between corresponding frames, the precision of corner point will be lower. In our previous study based on SURF points, we demonstrated the robustness of this method with an active background [6]. Thus, in this paper, SURF points will be adopted in the process of keypoint extraction. In the following step, we will establish the over determined equation via SURF feature points.

## The Process of Sub-Area Extraction

**Figure 2:**
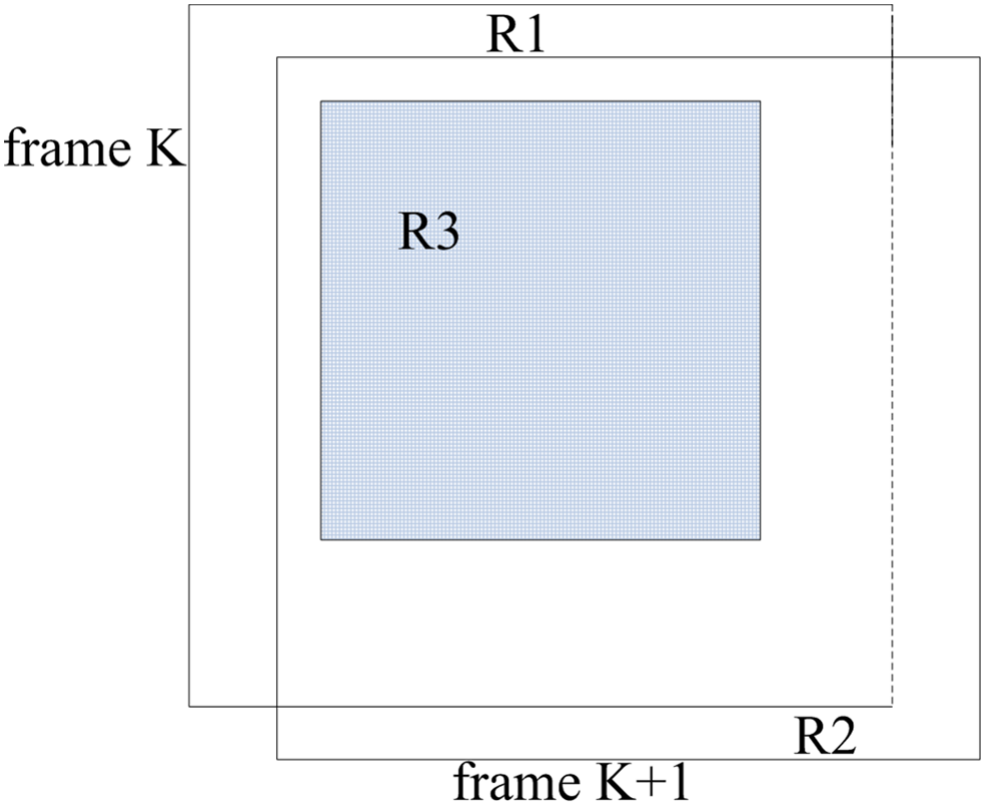
Relationship between relative frames.

Six transformation parameters will be calculated. In this paper, symbols a, b, c, d, e, and f stand for these motion parameters. Symbols a, b, c, and d represent the rotation and scale change relationships between corresponding frames. Symbols e and f represent translation transformation. Generally, we can find corresponding points in frame K+1 with respect to frame K. Certain unmatched points will occur in frame K+1 due to the motion transformation of camera. The relationship between these frames is shown in Figure 2.

In Figure 2, R1 represents the region in frame K disappearing in frame K+1. This region contains unmatched key points between relative frames. R2 shows the new area occurring in frame K+1. R3 represents the crossing region between relative frames, which will be extracted in the following step.

**Figure 3:**
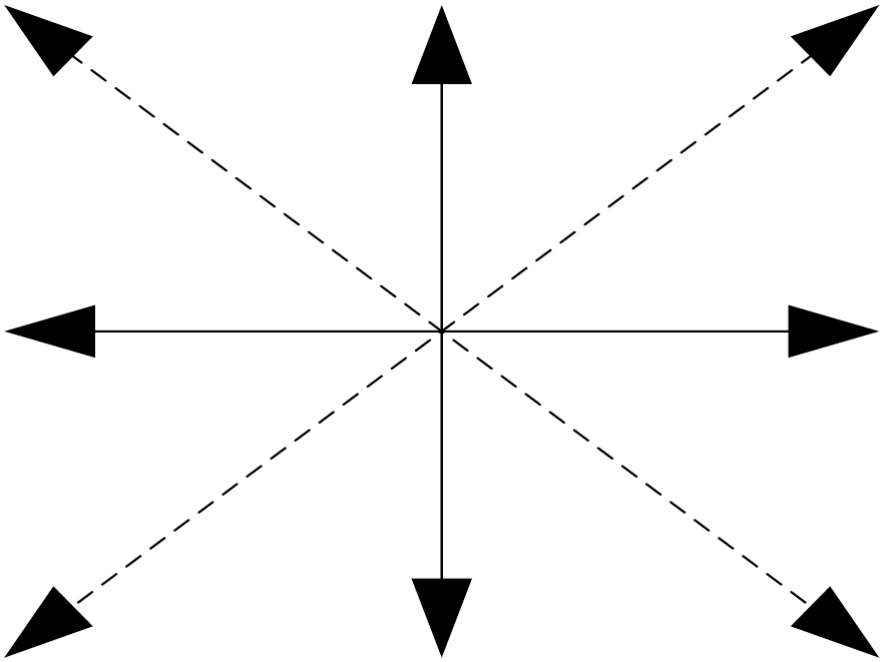
Motion directions of the camera.

After the previous steps, rotation and translation parameters will be obtained. According to these parameters, the origin point and maximum point will be introduced into the equation to localize the new positions of the origin point and maximum point in the following frame. On the basis of these results, the motion direction of the camera can be obtained. Figure 3 shows several directions. The dummy line represents the direction. Symbol e and parameter f represent translation transformation. The size of the region extracted between relative frames is 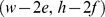, where 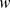 is the width of original image and 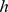 is the height of original image.

**Figure 4:**
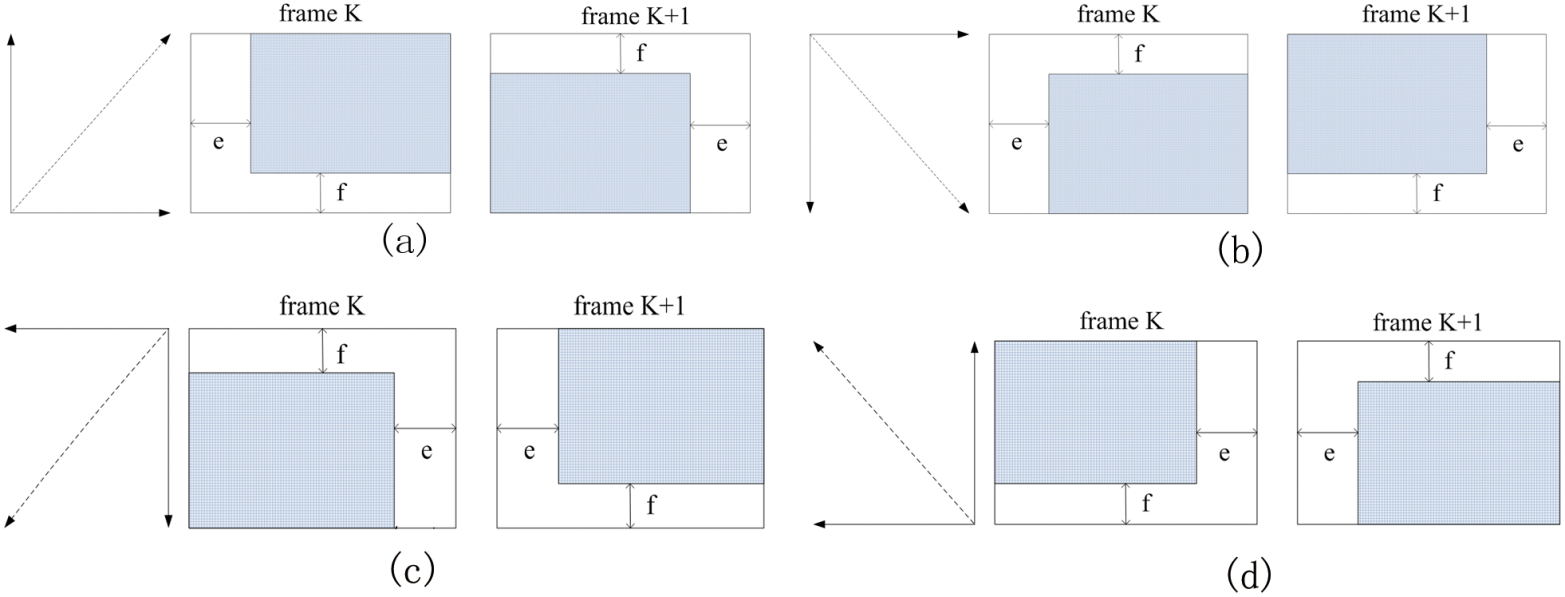
Several realignments with regard to the direction of the camera.

Figure 3 shows several motion directions of the camera. In the motion transformation model, the translation parameters obtained via calculations may not be the same results obtained via the two parameters motion model. Detailed explanations are below:

The plane coordinate is assumed to be in accordance with the default image location. The potential results are as follows:

1. In Figure 4(a), e>0 and f >0. The crossing region of relative frames is shown in Figure 4(a). We extract the sub-region in the top left of frame K. Meanwhile, we extract the relative area in the bottom right of frame K+1.
2. In figure 4(b), e>0 and f<0. Thus, we extract the area in the bottom right of frame K. Meanwhile, the corresponding region is extracted in the top right of frame K+1.
3. In Figure 4(c), e<0 and f<0. Thus, the sub-region will be extracted in the bottom right of frame K. Meanwhile, the corresponding sub-area will be extracted in the top right of frame K+1.
4. In Figure 4(d), e<0 and f>0. In this situation, we will extract the sub-region in the bottom right of frame K. In the meantime, the corresponding sub-area will be extracted in the bottom left of frame K+1.

Figure 4 shows the size of the extracted region in accordance with the vertical parameters and translation parameters. Our approach based on image matching and frame coupling makes full use of the relationship between the forward and backward frames, which does not mean the traditional relationship between frame K and frame K+1. In other words, the relationship does not focus on adjacent frames. Sometimes, the relationship between frame K and frame K+2 may be used. According to the practical application, we can establish the relationship between frame K and frame K+3. Based on this idea, our method detects an object via frame K. First, the extracted region will be selected as a template image. Then, this method implements image matching in frame K±i. After this procedure, we find the difference between two corresponding sub-areas. Before these procedures, the motion parameters of the camera have been obtained. Thus, we can adopt the whole region to perform image registration.

## Searching for Matching

The purpose of match searching is to select the best matching template image. The methods widely used in this realm include, the rhombus searching method, three steps searching method, and full searching method. All of these approaches begin by selecting the original searching location. Thus, the time consumed in each iteration step is not constant. If we select an inappropriate starting location, the method will need more steps, resulting in a low efficiency of the algorithm. Furthermore, inappropriate selection will not converge at the optimal solution.

**Figure 5:**
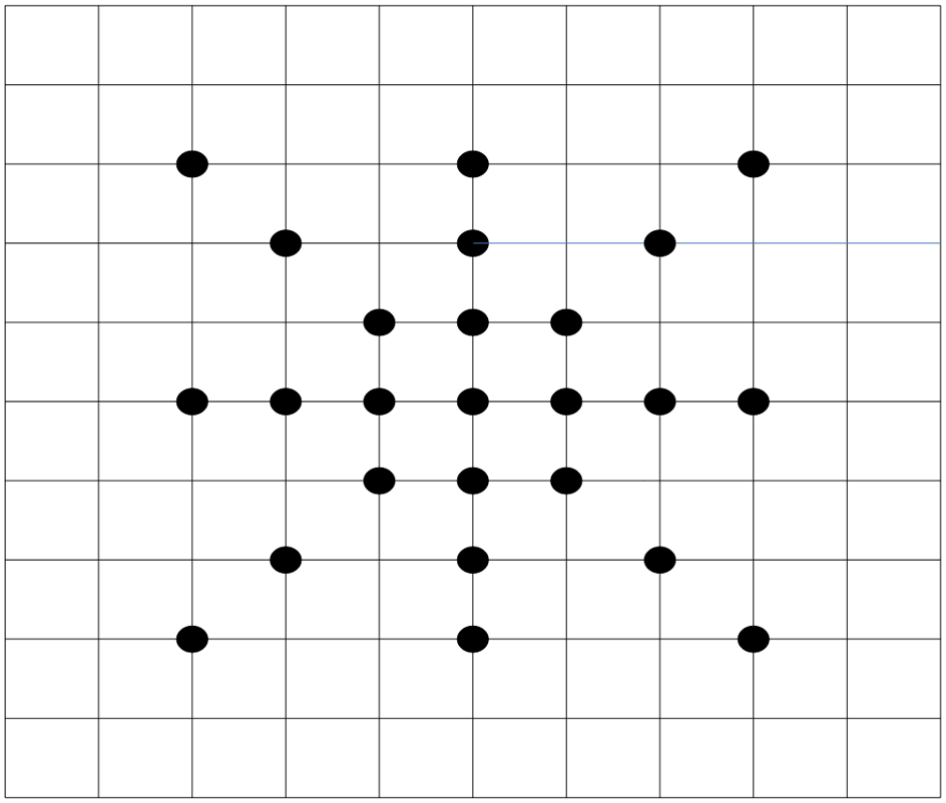
The sketch map of our proposed searching method.

The motion parameters of the camera will be the appropriate initialization with respect to the searching direction. Figure 4 shows the potential directions of the active camera. In this paper, we propose a new searching method with regard to the camera. This method can appropriately simulate the motion of the camera. Moreover, before starting this approach, the vertical parameters and horizontal parameters were obtained, which provide the potential initialization for this algorithm. To remedy the error from the calculation step, we implement the full searching process in four directions, which improves the reliability of our proposed approach. The sketch map of this method is shown in Figure 5.

Figure 5 shows that proposed method combines the merits of other searching approaches. It is distinct in that the region around the center point is the most appropriate matching area. Thus, we need to scatter more test points around the center point, which is the same process as the nine points searching method. Meanwhile, we scatter fewer test points in the region far from the center point, which is in accordance with the iteration searching method. The steps of our method are as follow:

### Step 1

According to the vertical and horizontal parameters obtained, we select a new template image T with size M.N. To improve the searching efficiency without interfering with the results, the size of the sub-area is a fixed value.

### Step 2

According to the obtained parameters, we can find the searching point-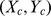 in frame K+1. Then, we scatter more test points around the center point. In other words, this method searches each point. Considering the combination with the rhombus searching method, the searching process is not complicated.

### Step 3

First, we calculate the similarity according to the vertical parameter and horizontal parameter. Then, we select the region with the least matching value as the BMV (Best Match Value). Meanwhile, we record the location of the best-matched sub-region, which is the best-matched area. In the application, we can enlarge the searching size when the values of the vertical and horizontal parameters are small. When the value exceeds the edge of the image, we cease this approach.

### Step 4

Then, we calculate other SAD values in other directions. Once the value is less than BMV, it is selected as BMV. Meanwhile, the best matching area will be refreshed.

### Step 5

Then, this method continues step 4 until all of the points are searched. In the application, the number of test points in one direction is 3. In selecting this number, the module value, according to vertical and translation parameters, should be considered. Sometimes, we have to add points. (Once this searching line touches the edge of frame K+2, the method stops).

Our proposed method reduces the time consumed in the iteration step according to several principles. With respect to area matching, the macro block is used to implement the process of matching, which reduces the time in matching section.

## Object Detection and Frame Coupling

On the basis of the relationship between the forward and backward frames, our method adopts image matching to detect the moving object. First, we select 3 forward frames as our templates. Meanwhile, 3 backward frames stand for the object to be searched. Then, we extract an area with a fixed size via the parameters obtained from the previous steps. Furthermore, we search for the matching region in the 3 backward frames. After the process, a matching area with the same size will be obtained.

**Figure 6:**
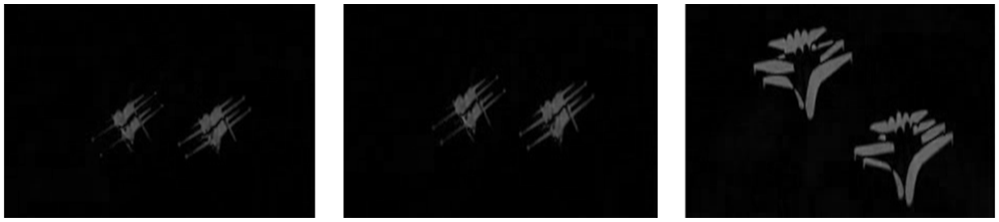
Difference images between adjacent matched frames.

Sub-region 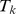 is assumed to be the extracted area from frame k. 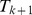
and 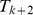 are the extracted sub-regions of frame K+1 and frame k+2, respectively. In searching for matching areas, 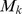 is the corresponding region of frame K. 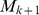 is the matched region of frame K+1. 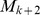 is the corresponding area of frame K+2. Scholars have proposed methods that detect the object by extracting the crossing region. However, these methods will cause other fringes in frames. Figure 6 shows the motion region obtained from the difference between adjacent frames. From the figure, we can find the fringes in frames, which do not meet the requirements of the practical applications.

Figure 6 indicates that the method based on adjacent image matching can obtain the potential region of the moving object. However, due to the fringes of the forward and backward frames, the results are not satisfactory. To remedy this drawback, the method combining frame coupling will be introduced into our proposed approach for detecting the object. The procedure is as follows:

First, the difference images are obtained for adjacent matching frames. Then, this method chooses the number of frames used for frame matching according to the complex extent of the background. In our research, the number of frames is 6. Thus, we can obtain 3 difference images from these matching frames. Moreover, this approach implements the process of frame coupling for the difference images obtained in the previous step. Finally, the moving object can be detected.

**Figure 7:**
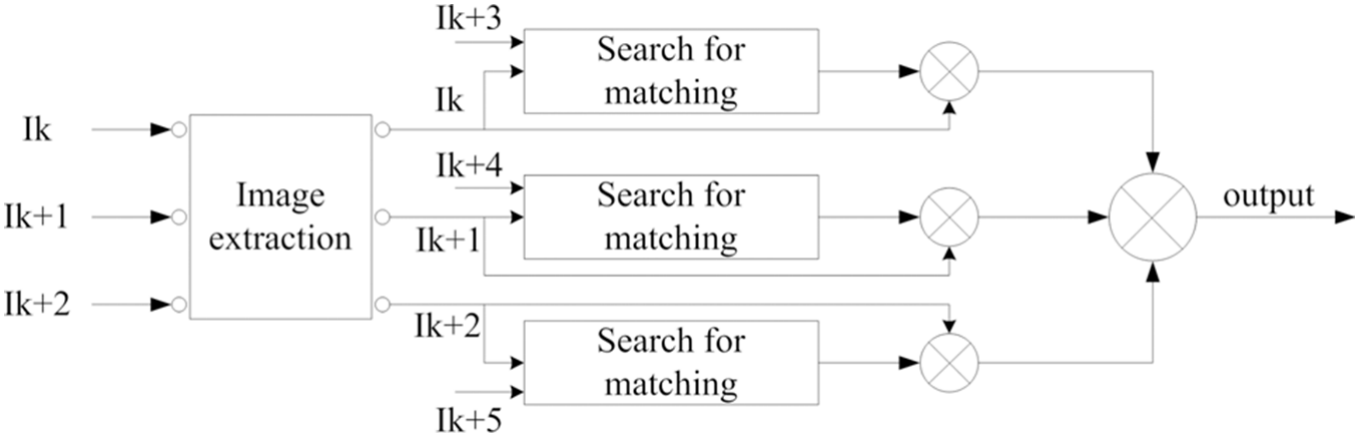
The framework of our algorithm for the cross-coupled detection method.



The process is shown in Figure 7. The calculation equations are shown in formula 1 and formula 2. 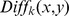, 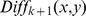, and 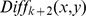 stand for the difference images between the template images and matching images. 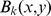 is the coupled image obtained from the difference images. Coupling is performed by comparing the results with T. When the values of these difference images are larger than 0, 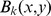 is set to 1. Otherwise, 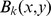 is 0. T is the threshold for binarization.

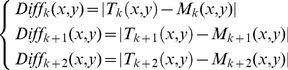
(1)

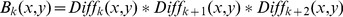
(2)

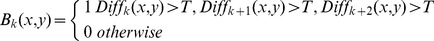
(3)


## The Object Detection Results

**Figure 8:**
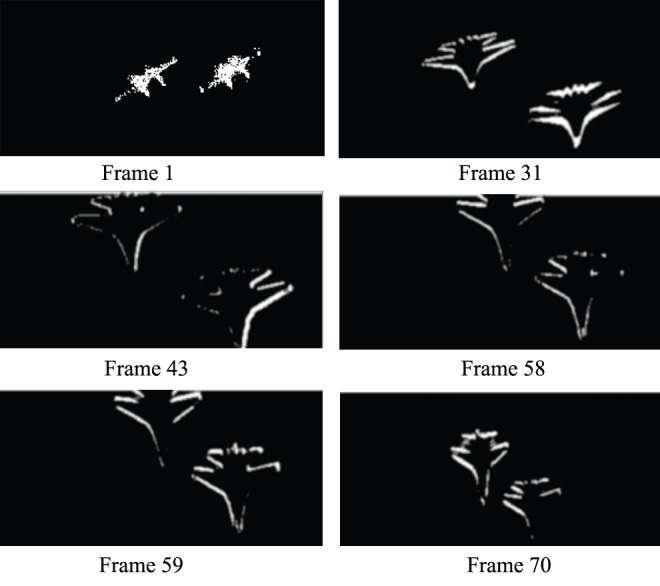
Object detection for image matching and coupling.

In order to validate the validity of the proposed methods, we choose a air show that includes multi-plane(multi-object), object moving posture variation and moving camera. The object detection results are shown in Figure 8, which show that the proposed method has good practicability for object detection under object moving posture variation and moving camera.

**Figure 9:**
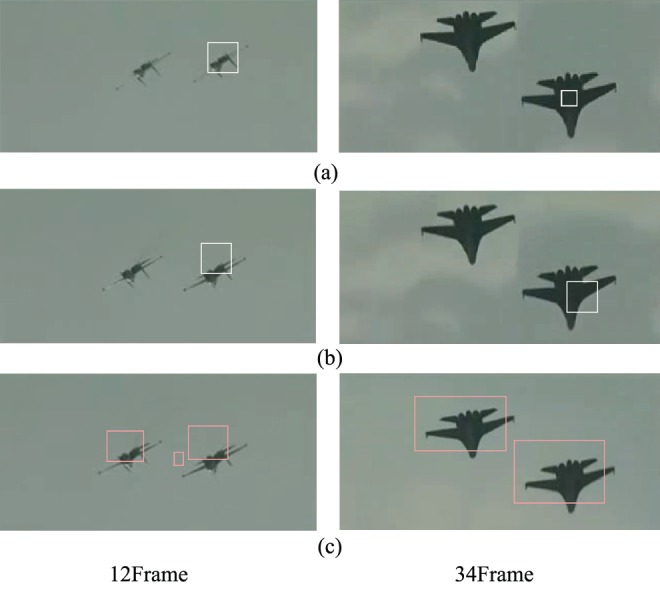
Object detection comparisons of three object detection algorithms.

Comparisons with three object detection algorithms (frame 59 and frame 70) are shown in Figure 9 and Table 1, which are feature points clustering, motion vectors histogram and image matching and frame coupling. From Figure 9, the feature points clustering and motion vectors histogram can’t detect two objects at the same time, because the two methods calculate the similarity and motion vectors histogram and from the most similar and the best match, so only get a similar region. Regarding on clustering method, when it acquires the match region, may get the best match region and the near-best mach region, so it takes some disturbances to object detection. Regarding on motion vectors histogram method, it is easily affected from outside interference and noise. While image matching and frame coupling is a contour extraction method from the whole, so long as there exist moving objects, this method can correctly detect them by frame coupling in the moving camera, but there is an extra dim small false object in Figure 9(c) 12. From Table 1, the fastest method is not image matching and frame coupling, so we will further optimize frame coupling method, which introduce couple control theory.

**Table 1:** Performance comparisons of three object detection algorithms.

	Feature Points Clustering	Motion Vectors Histogram	Image Matching and Frame Coupling
Multi objectsdetection	No	No	Yes
Accuracy rate	85%	90%	95%
Region integrity	Part	Part	Whole
Frames PerSecond	5	7	7

## Conclusion

In this paper, we present a new object detection method for image matching and coupling. This method adopts SURF points to obtain the motion parameters of the active camera. Then, this approach implements the process of image matching between frames. To improve the efficiency and accuracy, a new searching method is introduced. Then, to improve the accuracy of the potential object region, the method on coupling with frames is introduced into the detection section. Thus, we finish the process of object detection on the image and coupling. Experimental results demonstrate the feasibility and effectiveness of our proposed method, which provides a new object detection method for the moving camera.

## Supporting Information

Video S1 (AVI)
